# Curative and organ-preserving treatment with intra-arterial carboplatin induction followed by surgery and/or radiotherapy for advanced head and neck cancer: single-center five-year results

**DOI:** 10.1186/1471-2407-7-62

**Published:** 2007-04-11

**Authors:** Giulia Bertino, Marco Benazzo, Patrizia Gatti, Gianni Bernardo, Franco Corbella, Carmine Tinelli, Federico Zappoli, Eugenio Mira

**Affiliations:** 1 Department of Otolaryngology, University of Pavia – IRCCS Policlinico S Matteo, Pavia, Italy; 2 Department of Oncology, IRCCS Fondazione "Salvatore Maugeri", Pavia, Italy; 3 Department of Radiation Oncology, IRCCS Policlinico S. Matteo, Pavia, Italy; 4 Biometrics Unit, IRCCS Policlinico S. Matteo, Pavia, Italy; 5 Radiodiagnostic Unit, IRCCS Policlinico S. Matteo, Pavia, Italy

## Abstract

**Background:**

This study evaluated the feasibility, toxicity, response rate and survival of neoadjuvant superselective intra-arterial infusion of high dose carboplatin in advanced head and neck cancer.

**Methods:**

Forty-six patients with primary head and neck squamous cell carcinoma received 3 cycles of intra-arterial carboplatin (300 to 350 mg/m^2 ^per cycle every 2 weeks), followed by radiotherapy or surgery plus radiotherapy.

**Results:**

No complications or severe toxicity occurred. Sixteen patients (35%) were complete responders, 20 (43%) partial responders while 10 (22%) did not respond to treatment. After completion of the multimodality treatment, 38/46 patients (83%) were complete responders. After a 5-year follow-up period, 18/46 patients (39%) are alive and disease-free, 3 (6,5%) have died of a second primary tumor and 25 (54,5%) have died of the disease.

**Conclusion:**

Intra-arterial carboplatin induction chemotherapy is a safe, well-tolerated technique that discriminates between responders and non-responders and so may have prognostic significance in planning further integrated treatments aimed to organ preservation for advanced head and neck carcinomas.

## Background

In recent years chemotherapy has become an essential part of multimodality curative treatments for advanced head and neck cancer, with prolongation of survival and better organ preservation [1-3]. Several strategies have been developed to increase the efficacy of these integrated treatment protocols while decreasing their associated toxicity, e.g. induction or neoadjuvant versus concomitant chemotherapy, intra-arterial versus intravenous systemic administration of the drugs, single or multiple combined chemotherapeutic agents.

According to the detailed meta-analysis by Pignon et al. [1], today the gold standard therapy for patients with locally advanced head and neck squamous cell carcinoma seems to be concurrent chemotherapy-radiation treatment, with an absolute improvement of 8% in five year-survival compared to other protocols. However, some reports suggest that also induction chemotherapy may improve survival, locoregional control and organ preservation in oropharyngeal and laryngeal cancer [4-8]. According to Al Sarraf further advantages obtained by giving induction chemotherapy first, the incidence of systemic micrometastases is reduced, the cancer is downstaged in approximately 90% of the cases and up 50% of patients may achieve a complete clinical response [2].

A large body of evidence supports the theoretical attractiveness of intra-arterial chemotherapy, related to the first pass of the drug through the tumor bed and to the possibility of increasing the doses of the chemotherapeutic agent, thus minimizing systemic toxic side effects [9].

Compared to intravenous systemic chemotherapy protocols, intra-arterial regimens are based on the use of a single chemotherapeutic agent, namely cisplatin. Currently, platinum derivatives are the most effective drugs in the treatment of squamous cell carcinoma of the head and neck, and the second-generation platinum drug, carboplatin, possesses all of the radiopotentiation characteristics of cisplatin with less toxic side effects [2,10].

Over recent years our group has performed a pilot study of high-dose intra-arterial carboplatin induction chemotherapy on patients with untreated advanced squamous cell carcinoma of the upper aerodigestive tract. Neoadjuvant intra-arterial chemotherapy was part of a multimodality regimen combining radiotherapy in responder patients, surgery plus radiotherapy in resectable non-responder patients and palliative radiotherapy in non-resectable non-responder patients [11]. The purpose of the present study was to assess the feasibility and technical problems of intra-arterial transfemoral superselective perfusion, the maximum-tolerated dose of carboplatin, systemic and local toxicity and the response rate after induction chemotherapy on the same enlarged population after a longer term follow-up. After completing the therapeutic options built into our multimodality protocol, we assessed five-year overall and disease-free survival rates in patients.

## Methods

### Patient characteristics

Forty-six patients (43 men and 3 women), aged from 39 to 75 years (mean 58,3, median 42,1), with previously untreated squamous cell carcinoma of the upper aerodigestive tract were treated between November 1993 and December 2000. The pre-treatment characteristics of the patients and the site and stage of the tumour, according to the 4^th ^edition of the TNM used at that time [12], are reported in table 1 and table 2. The T2 patients were submitted to this protocol owing to general conditions that contraindicated surgery or because they refused surgery. Patients were required to sign informed consent approved by the Ethical Committee of the Institution.

### Pre-treatment evaluation

Patients underwent a complete clinical and laboratory examination including measurement of haematologic (complete blood cell and platelet count, prothrombin time, partial thromboplastin time), hepatic (blood bilirubin, transaminases and alkaline phosphatase level)and renal (blood urea nitrogen, creatinine and electrolytes level, urinanalysis, 24 hours creatinine clearance) parameters, as well as general metabolic functions (blood glucose and lipids level), chest radiography and cervical vessel ultrasonography. The disease was staged by physical examination, panendoscopic examination, biopsy, CT scan and/or MRI.

### Treatment protocol

All patients received intra-arterial (i.a.) infusions of carboplatin, dissolved in 80 mL of 0,9% saline and infused over 10–15 min by a battery-operated pump (Medrad Mark 5 Plus) with an infusion velocity of 6–8 mL/min. An angiographic catheter (Glidecath Radicofocus 5F Terumo) was introduced percutaneously under local anesthesia (10 mL of 2% lidocaine solution) into the common femoral artery, according to the Seldinger technique. The catheter tip was placed in the external carotid artery, under radiographic control. Diagnostic transfemoral carotid arteriography was performed and the most suitable branch of the artery, providing the main blood supply to the tumour, was selectively catheterised. The catheter was removed immediately after the infusion. A simultaneous intravenous (i.v.) infusion of 250 mL of 0,9% saline was performed. Dexamethasone (4 mg) and ondansetron (1 g) dissolved in 100 mL of 0,9% saline were administered i.v. 1 hour before the infusion. All patients received three cycles of intra-arterial chemotherapy, administered every two weeks. The planned dose of carboplatin per cycle was 300 mg/m^2 ^for the first cohort (3 cases) and 325 mg/m^2 ^for the second cohort (3 cases); subsequently, as no significant toxic effects were observed, the remaining patients (40 cases) received a dose of 350 mg/m^2 ^of carboplatin per cycle. Two weeks after completing chemotherapy, patients were restaged and subsequent treatment was decided upon on the basis of their objective response. Complete responders or partial responders had radiotherapy; non-responders with resectable disease underwent surgery on the tumor site and/or on the neck nodal metastases followed by radiotherapy; non-responders with unresectable disease underwent palliative radiotherapy. Radiation was delivered using conventional fractionation (1,8 – 2 Gy per fraction, five days per week). The total dose was 66 – 70 Gy for PTVI (planning target volume including tumour and encompassing all clinically positive nodes) and 50–56 Gy for PTVII (planning target volume including all anatomical regions at risk of subclinical malignant disease).

### Treatment evaluation

Toxicity was evaluated according to WHO criteria [13] 24 hrs after each drug administration. This included: assessment of haematologic, hepatic and renal parameters; assessment of gastrointestinal, otoneurological, cardiac and respiratory function, and the search for signs and symptoms of local toxicity. Response to therapy was assessed two weeks after the last administration of the drug by physical examination, endoscopy, imaging (CT scan and/or MRI) and biopsy, when necessary. A complete response (CR) was defined as the complete disappearance of all demonstrable lesions. A partial response (PR) was defined as a decrease of 50% or greater in the sum of the products of the largest perpendicular diameters of all measurable lesions. No response (NR) was defined as a regression of < 50% in total tumour size or as stable or progressive disease.

### Statistical analysis

The primary efficacy end point was overall survival (i.e, death as a result of any cause). Survival was measured from the start of treatment date to the date of death or the date when the patient was last known to be alive. Yearly estimates were calculated using the Kaplan-Meier method. Secondary efficacy end points were CR rates, loco-regional tumour control and disease-free survival. Time to loco-regional failure was measured from the start of treatment date and the date of disease relapse, the date of disease-related death, or the date when the patient was last known to be alive and disease-free. Loco-regional failure, distant metastases, or disease-related death were all considered failures for disease-free survival. Yearly disease-free survival rates were estimated with the Kaplan-Meier method.

Factors influencing overall and disease-free survival were analyzed and compared by the log-rank test. P values less than 0.05 were considered significant.

## Results

### Feasibility

One hundred and thirty eight super-selective transfemoral intra-arterial infusions of carboplatin were performed with no significant complications.

### Toxicity

Carboplatin treatment was well-tolerated by the majority of the patients. No instances of gastrointestinal (nausea, vomiting, diarrhoea), renal, cardiac, respiratory and otoneurological toxicity were reported. Cases of systemic and local toxicity are listed in table 3.

### Response rate

**Figure 1:**
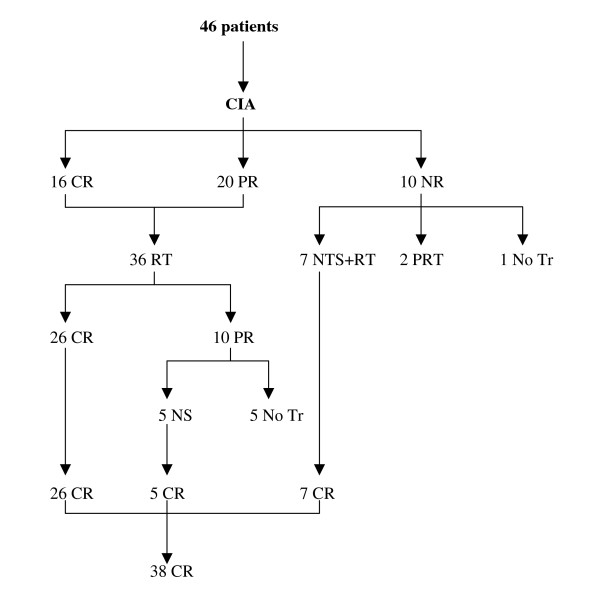
Locoregional response after completion of treatment protocol (CIA = intra-arterial chemotherapy; CR = complete response; PR = partial response; NR = no response; RT = radiotherapy; NS = neck surgery; NTS = primary and neck surgery; PRT = palliative radiotherapy; No Tr = no treatment).

After completing chemotherapy, 16 patients had a CR (35%), 20 patients had a PR (43%) and 10 patients had NR (22%). The 36 patients with a complete or partial response underwent radiotherapy, after which 26 patients achieved a complete response. Among the other 10 patients with persistent or recurrent disease in the neck, 5 underwent neck dissection and achieved a complete response and 5 refused any further treatment. Seven of the 10 NR patients underwent neck and primary site surgery, followed by radiotherapy. After this treatment, all 7 patients achieved a complete response. Of the remaining 3 non-responding patients, one refused any further treatment and two had palliative radiotherapy. Upon completing all treatments, 38/46 patients (83%) achieved a complete response: the 26 patients (CR) treated with induction chemotherapy and radiotherapy; the 5 (PR) treated with induction chemotherapy, radiotherapy and neck surgery and the 7 (NR) treated with induction chemotherapy, primary site and neck surgery and radiotherapy. (Fig. 1)

### Control of disease and survival

No patients were lost to follow-up. The median follow-up time, measured from the start of induction chemotherapy, was 51.3 months (range: 5 to 136 months).

**Figure 2:**
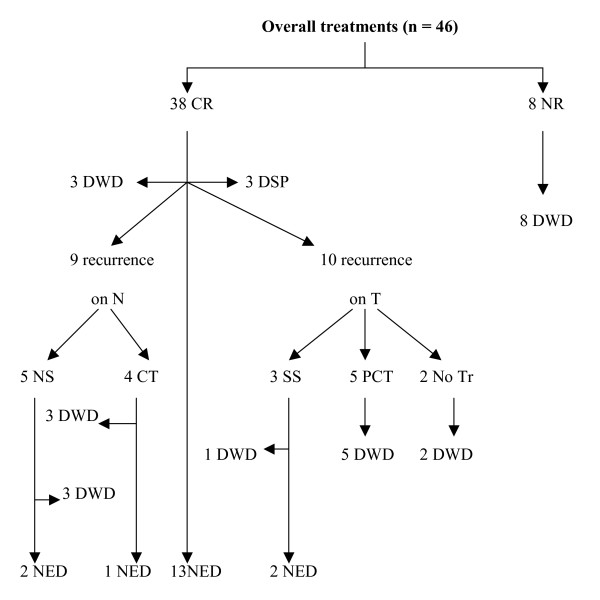
Clinical status of the patients at the end of follow-up (CR = complete response; PR = partial response; NED = no evidence of disease; DWD = death due to disease; DSP = death due to second primary tumour; NS = neck surgery; SS = salvage surgery; CT = systemic chemotherapy; PCT = palliative systemic chemotherapy; No Tr = no treatment).

The current status of the patients is as follows: after completing the treatment protocol, the 8 patients with persistent or recurrent disease (3 from the NR group and 5 from the PR group) have died of the disease; 13 of the 38 CR patients are alive and disease-free, 3 have died of distant metastases (2 pulmonary and 1 hepatic) and 3 due to a second primary tumour (2 lung cancers and 1 renal cancer), which occurred after a mean period of 29 months. The tumour recurred in 19 patients after a mean period of 25 months; 9 on the neck and 10 at the primary site. Five of the 9 patients with tumour recurrence on the neck underwent neck dissection; of these 2 are still alive and disease-free and 3 have died due to the persistence of the tumour; the other 4 received systemic chemotherapy. Of these 1 is alive and free of disease while 3 have died of the disease. Of the 10 patients in whom tumours recurred at the primary site, 5 underwent palliative systemic chemotherapy and died of the disease, 2 received only palliative medical treatment and died of the disease and 3 underwent salvage surgery. Of these, 2 are alive and disease-free and 1 has died of pulmonary metastases. In conclusion, after a 5-year follow-up, 18 patients (39%) are alive and disease-free, 3 (6,5%) have died due to a second primary tumour and 25 (54,5%) have died of the disease (Fig. 2).

The percentage of organ preservation was 83%, as 8 of the 46 patients underwent salvage surgery at the tumour site.

**Figure 3:**
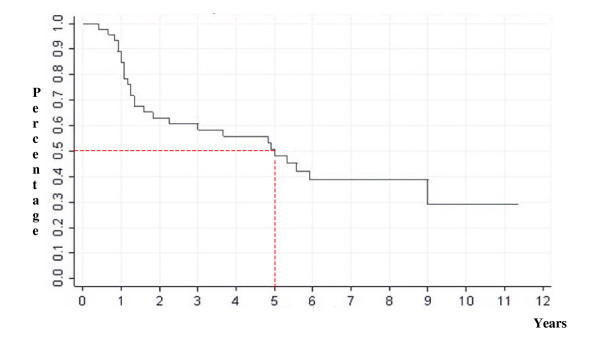
Overall survival of 46 patients.

**Figure 4:**
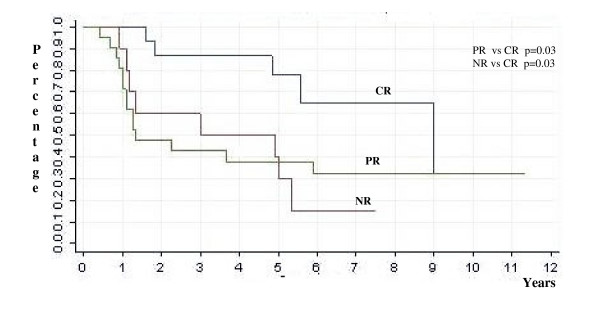
Overall survival according to the response to induction chemotherapy (CR = complete response; PR = partial response; NR = no response).

The probability of five-year overall survival was 50% (Fig. 3). The main factor influencing overall survival was the response to induction chemotherapy: CR patients showed statistically significant better survival rates compared to PR or NR patients, even if 12 (40%) of these patients were successfully submitted to salvage surgery (Fig. 4).

**Figure 5:**
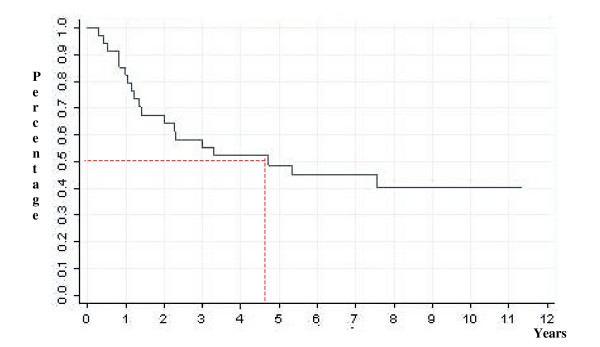
Disease-free survival of 38 patients.

**Figure 6:**
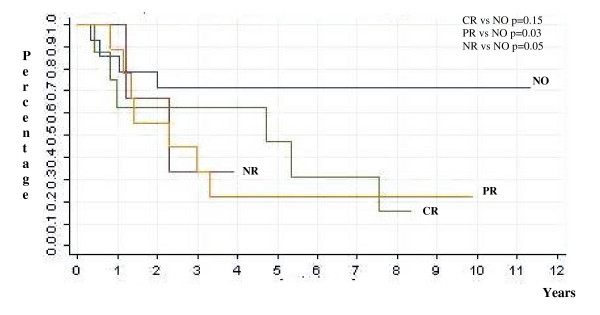
Disease-free survival according to neck status (NO = no lymphnodes at diagnosis; CR = complete response; PR = partial response; NR = no response).

Five-year disease-free survival, calculated on the 38 patients who were free of disease after completing all treatments, was 49,8% (Fig. 5). In this case, the main factor influencing disease recurrence or the appearance of distant metastases was patients' neck status: patients with no metastatic lymph-nodes or patients whose metastatic lymph-nodes regressed completely after induction chemotherapy had a better disease-free survival rate than patients with positive nodes who had a partial or no response to chemotherapy (Fig. 6).

## Discussion

Our present data confirmed the evidence that regional chemotherapy by superselective transfemoral infusion of high doses of platinum compounds is feasible and that, with the cooperation of an experienced interventional radiologist, can be easily reproduced in a multi-institutional setting, with minimal procedural complications [9,11,14-16].

The advantage of carboplatin is that it has an antitumour activity comparable with that of cisplatin but a toxicology profile with lower renal and gastrointestinal effects [10,17-19]. We administered far higher doses of carboplatin than those used by Vieitez et al. [15], according to our preliminary phase I study [20] that led us escalating the cycle doses of carboplatin from 300 mg/m2 to 350 mg/m2. These doses were comparable to the cisplatin "decadoses" used by Robbins et al. [21-24], without relying on simultaneous i.v. treatment with sodium thiosulphate to achieve systemic neutralisation of the platinum compound.

Systemic toxicity was low in our study population: all patients completed the three cycles of chemotherapy with no chemotherapy-related deaths, only 4 cases of moderate (grade 1–2) hematologic toxicity, and one case each of high (grade 3–4) hepatic and neurological toxicity. Local toxicity occurred in some cases, mainly at the level of skin and oral mucosa, but of short duration and of low/moderate degree. The higher systemic toxicity observed in other studies based on the same intra-arterial chemotherapy approach could be due to the different activity/toxicity profile of cisplatin versus carboplatin or to the potentiation of toxic effects in concurrent chemotherapy-radiotherapy regimens.

With respect to a classic neoadjuvant intravenous chemotherapy protocol (Al Sarraf) our protocol presents the advantage of a reduced systemic toxicity and of a reduced duration (one day vs. three days administration, two weeks vs. three weeks interval), with a significant improvement of patient's compliance.

A true neoadjuvant intra-arterial chemotherapy approach for head and neck cancer treatment is rare and the results of many trials are difficult to compare, owing to a number of methodological differences [16]. Limiting our comparison to the latest studies conducted with platinum compounds, our response rates at the end of induction chemotherapy (CR 35% and PR 43%) are similar to those observed by Kovacs [16] (CR 38% and PR 31%) and lower than those observed by Wilson et al [25] (CR 68% and PR 23%).

Our five-year overall and disease-free survival rates (50% and 49,8%) may be compared with those observed by Kovacs [16] (77% and 59%). Even if concurrent chemoradiation treatments seem to achieve the best overall survival rate [1], our results (50% and 49,8%) are similar to those obtained with the RADPLAT protocol (38,8% and 53,6%) [26].

According to our previous results [11] and the opinion of other authors [2,27], we consider the response to induction chemotherapy to be an important prognostic factor which may determine the sequence and timing of further planned definitive therapy: patients with a lesser response will require total surgical resection of the disease or the remaining disease, followed by post-surgical radiation therapy; patients with a good response may avoid surgery and be treated with radiotherapy alone. Therefore, on the basis of the evaluation made after induction chemotherapy, we adopted a flexible protocol, based not only on the patient's compliance and general conditions, but mainly on their response to neoadjuvant chemotherapy. A similar but less customized multimodality treatment has been followed by Kovacs et al. [28] in a large series of oral and oropharyngeal cancer patients.

## Conclusion

Our present experience suggests that in the treatment of head and neck cancer, superselective intra-arterial infusion with high doses of carboplatin delivered directly to the tumour bed is a safe and well-tolerated technique with no major systemic or local toxic side effects. It proves once again that, compared to other protocols (e.g. concurrent chemoradiotherapy), systemic or intra-arterial induction chemotherapy, does not have a significant advantage in terms of overall or disease-free survival. However, in some patients with bulky head and neck tumours, this technique, adopted as part of a first-line multidisciplinary approach, can achieve a high response rate, which may have a prognostic significance. This is a prospective study of a cohort of patients treated in a unique fashion in which carboplatin was given as an intra-arterial infusion. Those patients who had a major response were than treated with radiation therapy only. Non-responders were treated with surgery. The protocol introduces a novel algorithm for management of patients with advanced head and neck cancer. It is designed to triage patients who are sensitive to chemotherapy to non-surgical regimen whereas those who are not sensitive are treated with surgery.

## Competing interests

The author(s) declare that they have no competing interests.

## Authors' contributions

GB and PG prepared and edited the manuscript. MB, FC, GB and FZ were involved in revising results and in preparation of the different topics of the manuscript (surgery, radiotherapy, chemotherapy and infusional technique). CT performed the statistical analysis. EM gave the final approval of the version of the manuscript.

## Pre-publication history

The pre-publication history for this paper can be accessed here:



## Figures and Tables

**Table 1:** Pre-treatment characteristics of the patients

**Patient characteristics**	**Number**
*Sex*	
Males	43
Females	3
*Age*	
Range	39–75
Mean	58.3
Median	42.1
*Performance status*	
(Karnofsky scale)	> 80
*Tumor stage*	
II	7
III	15
IV	24
*Primary tumour site*	
Oral cavity	9
Oropharynx	28
Hypopharynx	3
Nasopharynx	1
Oral cavity + Oropharynx	4
Oropharynx + Hypopharynx	1

**Table 2:** Pre-treatment tumour (T) and lymph node (N) staging

	**N0**	**N1**	**N2a**	**N2b**	**N2c**	**N3**	**TOTAL**
**T2**	8	4	5	1	1		19
**T3**	9	5	3	1	1		19
**T4**	1	4		2	1		8
**TOTAL**	18	13	8	4	3	0	46

**Table 3:** Toxicity from chemotherapy

**Type of toxicity**	**Grade**	**N. of patients**
Neutropenia	2	2
Thrombocytopenia	1	2
Hyperbilirubinemia	4	1
Peripheral neuropathy	3	1
Alopecia	1–3	1
Stomatitis	2–3	2
Skin rash	3	1
